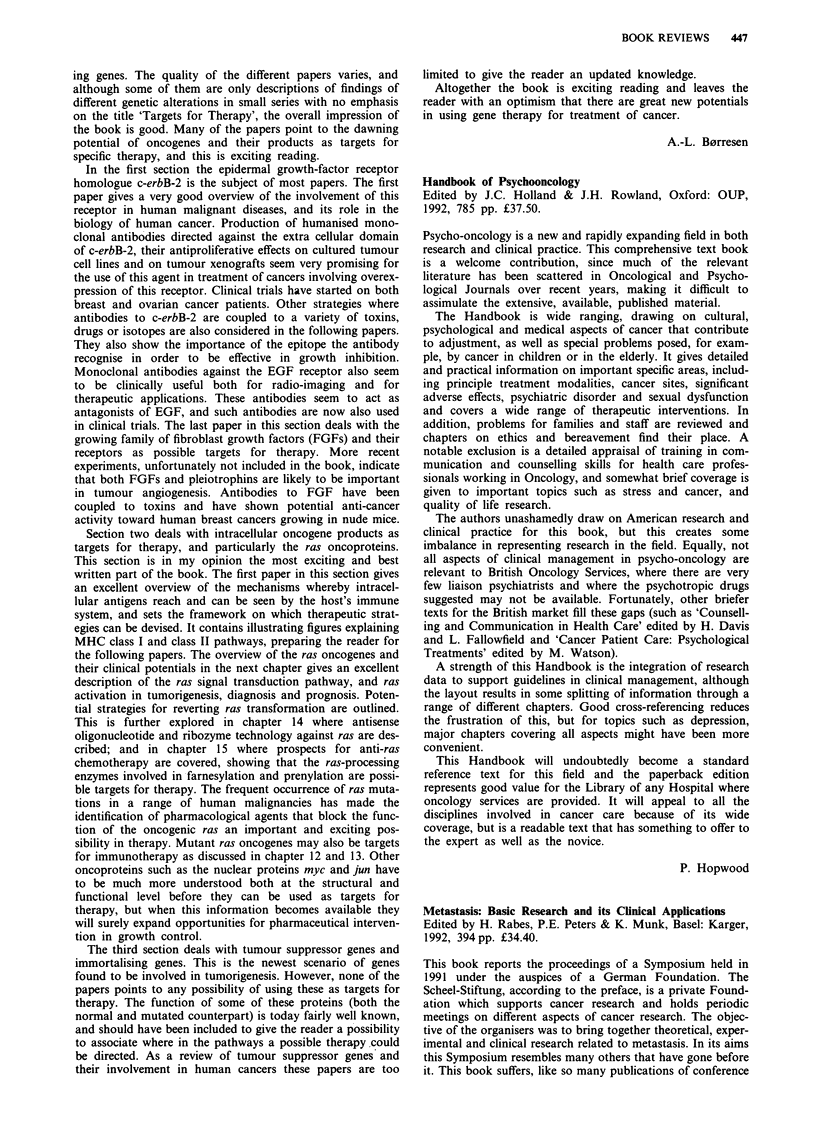# Handbook of Psychooncology

**Published:** 1993-08

**Authors:** P. Hopwood


**Handbook of Psychooncology**
Edited by J.C. Holland & J.H. Rowland, Oxford: OUP, 1992, 785 pp. £37.50.

Psycho-oncology is a new and rapidly expanding field in both research and clinical practice. This comprehensive text book is a welcome contribution, since much of the relevant literature has been scattered in Oncological and Psychological Journals over recent years, making it difficult to assimulate the extensive, available, published material.

The Handbook is wide ranging, drawing on cultural, psychological and medical aspects of cancer that contribute to adjustment, as well as special problems posed, for example, by cancer in children or in the elderly. It gives detailed and practical information on important specific areas, including principle treatment modalities, cancer sites, significant adverse effects, psychiatric disorder and sexual dysfunction and covers a wide range of therapeutic interventions. In addition, problems for families and staff are reviewed and chapters on ethics and bereavement find their place. A notable exclusion is a detailed appraisal of training in communication and counselling skills for health care professionals working in Oncology, and somewhat brief coverage is given to important topics such as stress and cancer, and quality of life research.

The authors unashamedly draw on American research and clinical practice for this book, but this creates some imbalance in representing research in the field. Equally, not all aspects of clinical management in psycho-oncology are relevant to British Oncology Services, where there are very few liaison psychiatrists and where the psychotropic drugs suggested may not be available. Fortunately, other briefer texts for the British market fill these gaps (such as 'Counselling and Communication in Health Care' edited by H. Davis and L. Fallowfield and 'Cancer Patient Care: Psychological Treatments' edited by M. Watson).

A strength of this Handbook is the integration of research data to support guidelines in clinical management, although the layout results in some splitting of information through a range of different chapters. Good cross-referencing reduces the frustration of this, but for topics such as depression, major chapters covering all aspects might have been more convenient.

This Handbook will undoubtedly become a standard reference text for this field and the paperback edition represents good value for the Library of any Hospital where oncology services are provided. It will appeal to all the disciplines involved in cancer care because of its wide coverage, but is a readable text that has something to offer to the expert as well as the novice.

P. Hopwood